# Physical therapy as a promising treatment for osteoarthritis: A narrative review

**DOI:** 10.3389/fphys.2022.1011407

**Published:** 2022-10-14

**Authors:** Wei Wang, Yonggang Niu, Qingxiu Jia

**Affiliations:** ^1^ School of Physical Education, Anyang Normal University, Anyang, China; ^2^ Anyang Key Laboratory of Fitness Training and Assessment, Anyang Normal University, Anyang, China

**Keywords:** osteoarthritis, exercise, physical therapy, rehabilitation medicine, OA

## Abstract

Osteoarthritis (OA) is the most prevalent joint disease and a leading cause of disability in older adults. With an increasing population ageing and obesity, OA is becoming even more prevalent than it was in previous decades. Evidence indicates that OA is caused by the breakdown of joint tissues from mechanical loading and inflammation, but the deeper underlying mechanism of OA pathogenesis remains unclear, hindering efforts to prevent and treat this disease. Pharmacological treatments are mostly related to relieving symptoms, and there is no drug for radical cure. However, compelling evidence suggests that regular practice of resistance exercise may prevent and control the development of several musculoskeletal chronic diseases including OA, which may result in improved quality of life of the patients. In this review, we introduced the current understanding of the mechanism and clinical treatments of OA pathogenesis. We also reviewed the recent study of physical therapy in the treatment of skeletal system disorders, especially in OA. Finally, we discuss the present challenges and promising advantages of physical therapy in OA treatment.

## 1 Introduction

Osteoarthritis (OA) is a common chronic joint disease characterized by articular cartilage erosion, osteophyte formation, subchondral sclerosis, and a series of biochemical and morphological changes in the synovium and articular cavity (Hunter and Bierma-Zeinstra, 2019; Abramoff and Caldera, 2020; Zhang et al., 2020). Due to the ageing of the population, unhealthy diets, changed lifestyles and increased obesity, the incidence of OA is gradually increasing, and approximately 7% of the global population suffers from OA (Hunter et al., 2020). OA can seriously affect the quality of life and is one of the main causes of adult disability (Quicke et al., 2022). OA has long been considered a disease of cartilage degeneration that can be effectively treated surgically at severe stages by knee and hip replacements (Gee and Lee, 2012; Roos and Arden, 2016; Kolasinski et al., 2020). In recent years, OA has gradually been considered a whole-organ disease with multiple risk factors that develops slowly for many years (Hussain et al., 2016). Joint injury, genetic predisposition, biomechanics, metabolic syndrome and gut microbiome are all modifiable risk factors for OA (Berenbaum et al., 2018). Thus, early prevention and comprehensive treatments are preferred. In addition, the early drug treatments of OA mainly aim to relieve pain, delay disease progression and protect joint function, but there are nearly no effective disease-modifying drugs (Cao et al., 2020; Latourte et al., 2020). The complex pathogenesis of OA and the unclear underlying mechanism hinder the development of new drugs or treatments. Therefore, it is very important to clarify the underlying mechanism of its pathogenesis and discover new approaches to prevent and treat OA.

Physical therapy is the most commonly recommended nonpharmacological and nonsurgical treatment for musculoskeletal diseases, especially OA (Rausch Osthoff et al., 2018; Kolasinski et al., 2020). Physical therapy is economical and convenient, with few or minor adverse reactions (Fritz et al., 2015; Rhon et al., 2022). The goal of physical therapy for OA is to reduce pain, improve joint function and improve the patient’s physical condition, enabling the patient to gain sufficient mobility in activities of daily living. Previous studies have demonstrated the great benefits of physical therapy for OA (Kovar et al., 1992; Deyle et al., 2000; Deyle et al., 2005; Rhon et al., 2022). The paradigms of physical therapy for OA patients mainly include aerobic exercise, resistance training, acupuncture, yoga and Tai Chi. However, at present, the effectiveness of physical therapy on OA is not fully understood, let alone the mechanism by which physical therapy promotes OA repair. Moreover, the unified standards for the indications, contraindications, optimal treatment parameters and optimal course of physical therapy in clinical practice hamper the clinical practice and effectiveness of physical therapy and lead to inconsistencies in the current clinical trials from different groups. Therefore, more preclinical and clinical studies are needed to determine the effect of physical therapy on OA and the underlying mechanism, which can help to put forward new treatment schemes.

We searched PubMed for English-language articles on physical therapy for OA, using the search terms physical therapy, physical therapy and osteoarthritis, osteoarthritis and treatment; osteoarthritis and epidemiology; osteoarthritis and diagnosis; osteoarthritis and risk factors; physical therapy and patient education; and physical therapy and exercise models. We reviewed these publications and relevant references. In this review, we first introduced the prevalence and clinical symptoms of OA, the current understanding of the risk factors and pathogenesis mechanism of OA, and its current clinical treatments. We also reviewed the recent study of physical therapy in the treatment of skeletal system-related disorders, especially OA. Finally, we discuss the present challenges and future improvements of physical therapy in the treatment of OA.

## 2 Pathogenesis and treatments of osteoarthritis

OA is a common chronic joint disease with multiple pathogenic factors that can seriously lower the quality of life without disease-modifying medications. For decades, the number of OA patients has gradually increased, with a trend of younger age. Thus, a comprehensive understanding of OA is important for patients, researchers, and clinicians.

### 2.1 Prevalence and symptoms of osteoarthritis

OA is a highly prevalent chronic joint degenerative disease, and approximately 7% of the global population suffered from OA according to a 2020 study (Hunter et al., 2020) with over 300 million people worldwide (Collaborators, 2018). In addition, Ian et al. (Wallace et al., 2017) reported that knee OA has doubled in prevalence since the mid-20th century with the increases in life expectancy. OA has long been considered a degenerative disease of the aged; however, its development starts much earlier than originally thought (Roos and Arden, 2016). In addition, the prevalence of OA increases not only because of longer life expectancy but also because of change in modern lifestyle, particularly diets rich in sugar and saturated fats, leading to chronic low-grade inflammation and obesity (Mobasheri et al., 2017; Berenbaum et al., 2018; Plotz et al., 2021). Thus, OA is becoming more common at a younger age and ranks among the top 20 diseases in the 40- to 45-year-old group (Evaluation, 2015). Recent data from Turkiewicz et al. (2014) estimated an increase in OA from 26.6% in 2012 to 29.5% in 2032 among those aged 45 years and older.

### 2.2 Typical symptoms of osteoarthritis

**FIGURE 1:**
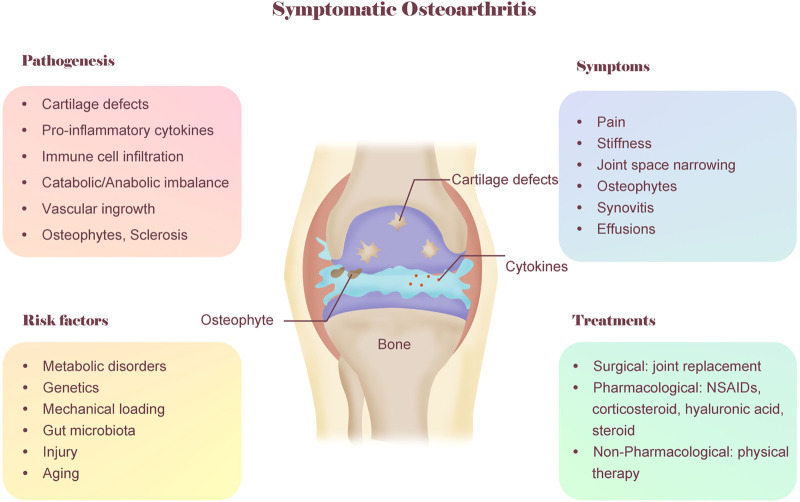
A conceptual model of OA including pathogenesis, symptoms, risk factors and treatments. OA is considered as a whole-organ disease and involves multiple factors. The typical symptoms of OA are pain and stiffness. The typical features of OA noted on radiographs include joint space narrowing and osteophytes. The pathogenesis of OA is complex, involving cartilage defects, inflammation, metabolic imbalance, etc. OA also involves multiple risk factors, including genetics, metabolic disorder, biomechanics, aging and gut microbiota. The current treatment for OA mainly include three categories: surgical, pharmacological, non-pharmacological. As a non-pharmacological treatment, physical therapy is promising for treating OA patients due to its cost-saving, convenience and few adverse reaction.

The typical symptoms of OA patients are pain and stiffness in the joint (Figure 1). The pain is usually provoked by load bearing and relieved by rest, but it may become less predictable over time. Stiffness is worse in the morning or on arising after prolonged sitting (Metcalfe et al., 2019; Yu et al., 2022). The typical features of OA noted on radiographs include joint space narrowing with degradation of articular cartilage and meniscus, as well as bony changes, including sclerosis of subchondral bone and osteophytes (Kraus et al., 2015; Katz et al., 2021). Due to the damaged tissues in OA joints, synovitis occurs, which is common in OA patients and is triggered by the macrophage-mediated innate immune response (Zhang et al., 2018; Wood et al., 2019). In addition, on physical examination, knee effusions of OA patients are generally either absent or small and cool. However, other arthritis patients, such as rheumatoid arthritis patients, often have warm, easily palpable effusions (Kraus et al., 2015). The comprehensive understanding of OA symptoms helps clinicians to distinguish OA from other diseases that can cause joint pain.

### 2.3 Risk factors and mechanism of osteoarthritis pathogenesis

Although OA is directly caused by articular cartilage degeneration and acute injury, the aetiology of OA is complex and involves multiple factors (O'Neill et al., 2018), including genetic predisposition, metabolic syndrome, biomechanics, chronic inflammation, and gut microbiota (Figure 1).

#### 2.3.1 Metabolic disorders

Increasing clinical studies suggest that metabolic syndromes, such as obesity and diabetes, play a role in the incidence and progression of OA (Berenbaum et al., 2017; Mobasheri et al., 2017). Obesity is usually caused by unhealthy diets (high fat or high glucose) and physical inactivity, which is a major reason for the increasing population of OA. According to the study from Reyes et al. (2016), overweight and obesity could increase the risk of hand, hip, and knee OA among adults aged ≥40 years in Spain, especially for the knee OA. They reported that overweight and (grade I, II) obesity increased knee OA risk by a factor of 2-, 3.1- and 4.7-fold, respectively, compared with healthy weight. Chen et al. (2020a) reported that 57.58% of patients in Australia who had knee replacement were affected by obesity. Patients with overweight, obesity class I and II or obesity class III received a knee replacement surgery 1.89, 4.48 and 8.08 years earlier than patients with normal weight, respectively.

Obesity is usually associated with insulin resistance and type 2 diabetes mellitus. The incidence of arthroplasty was approximately 3 times higher in patients with type 2 diabetes than in those without, along with more severe clinical symptoms of OA (Schett et al., 2013). Chen et al. (2017) reported that subchondral bone remodelling led to deteriorated microstructure and strength, which in turn aggravated cartilage degradation in type 2 diabetes patients. In a mouse model, Wang et al. (2021) also found that diabetes mellitus accelerates the progression of osteoarthritis in streptozotocin-induced diabetic mice by deteriorating subchondral bone microarchitecture. Using a mouse model with spontaneously hypertensive heart failure and obesity, Deng et al. (2018) evaluated the contribution of metabolic syndrome to OA development, which exhibited knee joints with severe loss of the entire thickness of the cartilage associated with pronounced osteophyte formation and fibrosis.

High levels of dietary cholesterol are also considered to be associated with the pathogenesis of multiple diseases, including OA (Song et al., 2021). Choi et al. (2019) provided the direct evidence that chondrocytes in OA mice had increased levels of cholesterol because of enhanced uptake, upregulation of cholesterol hydroxylases and increased production of oxysterol metabolites. Notably, poor diet and obesity would not only affect ourselves, but also have a negative effect on our offspring. Guilak’s group (Harasymowicz et al., 2020) reported that parental high-fat feeding showed an intergenerational effect on the inheritance of increased metabolic imbalance and injury-induced OA in mice. Thus, maintaining a healthy diet and good body metabolism are very important in the prevention and treatment of OA.

#### 2.3.2 Genetics

Osteoarthritis has long been thought to have a strong genetic correlation (Collaborators et al., 2012; Tachmazidou et al., 2019). Studies from two groups reported a functional SNP in the 5′ UTR of GDF5 and a functional polymorphism in the 5′ UTR of GDF5 associated with osteoarthritis (Miyamoto et al., 2007; Chapman et al., 2008). The GWAS from Kingsley’s group reported that an ancient regulatory variant in a novel growth enhancer (GROW1) has been repeatedly selected in northern environments and explained the high frequency of a GDF5 haplotype that increases arthritis susceptibility (Capellini et al., 2017). Unnur et al. (Styrkarsdottir et al., 2017) also discovered two rare signals that are strongly associated with osteoarthritis total hip replacement: a missense variant in the COMP gene and a frameshift mutation in the CHADL gene. In addition, a common missense variant in the COL11A1 gene, a variant of CHADL1, is also associated with hip osteoarthritis (Styrkarsdottir et al., 2018). Daniel et al. (Richard et al., 2020) revealed selection and constraint on knee regulatory elements, including those overlapping osteoarthritis risk variants, by epigenetic profiling of chondrocytes and discovered a causal enhancer variant present at a risk locus (GDF5-UQCC1). Cindy et al. (Boer et al., 2021) conducted a GWAS meta-analysis across knee, hip, finger, thumb, and spine osteoarthritis phenotypes in 826,690 individuals and identified sex-specific and early age-at-onset osteoarthritis risk loci.

#### 2.3.3 Mechanical loading

Although joints are essential for load bearing and articular cartilage is constantly challenged by mechanical stress, aberrant mechanical load could be one of the primary aetiological factors that leads to cartilage injury (Hodgkinson et al., 2022). Chang et al. (2019) identified gremlin-1 as a mechanical loading-inducible factor in chondrocytes that activated nuclear factor-κB signalling and induced of catabolic enzymes. Zhang et al. (2022) reported that mechanical overloading accelerated senescence in mouse articular cartilage and that FBXW7 is a key factor in the association between mechanical overloading and cartilage ageing in OA. Zhen et al. (2021) reported that high mechanical stress stimulates transforming growth factor beta (TGFβ) activity and that a high level of TGFβ disrupts cartilage homeostasis and impairs the metabolic activity of chondrocytes. Whasil et al. (Lee et al., 2014) identified Piezo1 and Piezo2 mechanosensitive ion channels in chondrocytes as transduction channels for high-strain mechanical stress. Their recent study further discovered that osteoarthritis-relevant levels of interleukin-1α reprogrammed articular chondrocytes and upregulated Piezo1 expression, leading to a Ca2+-driven feed-forward mechanism that underlies the progression of OA (Lee et al., 2021).

#### 2.3.4 Gut microbiota

In recent years, the gut microbiota has been considered as an essential component of body health. In addition, the gut microbiota has also caught the attention of medical experts in OA pathogenesis (Huang and Kraus, 2016; Biver et al., 2019; Liu et al., 2019). For instance, in a population-based study, Wei et al. (2021a) provided the first evidence of alterations in the composition of the gut microbiome in OA patients. They reported that a low relative abundance of Roseburia but a high relative abundance of Bilophila and Desulfovibrio at the genus level were associated with prevalent symptomatic hand OA. Christopher et al. (Dunn et al., 2020) also revealed a microbial DNA signature in human and mouse cartilage and found increased Gram-negative constituents during the progression of OA. Huang et al. (2020) reported the correlation of a greater abundance of *Fusobacterium* and *Faecalibacterium* and a lesser abundance of Ruminococcaceae with OA severity. Cindy et al. (Boer et al., 2019) also found a significant association between *Streptococcus* species abundance in stool microbiome samples, knee pain and knee inflammation. These studies established a direct gut microbiome-OA connection and indicated that the microbiome is a possible therapeutic target for OA.

#### 2.3.5 Gender and sex

Gender and sex differences are usually ignored when discussing the risk factors for OA. However, emerging evidence has shown the differences in the incidence and severity of OA between male and female patients. Virtually, there is a greater age-adjusted prevalence in female patients than in male patients (Boyan et al., 2012; Barbour et al., 2017) and female patients usually suffer more from OA than males (Laitner et al., 2002; Tschon et al., 2021). A meta-analysis from Srikanth et al. (2005) showed that males had a greater risk of cervical spine OA, but females, particularly after menopausal age, tended to have a higher incidence of knee OA with more severe symptoms. The pathogenesis of OA can also be differential between males and females. For example, Liao et al. (2022) reported that genetic ablation of interleukin-6 in male mice decreased cartilage degradation, and nociceptive innervation in a posttraumatic OA mouse model. However, this effect was sex-specific because it was not observed in female mice. A recent study from Xu et al. (2022) also showed that the predominantly expressed MAPK signalling pathway and the thyroid hormone signalling pathway were more highly expressed in postmenopausal women than in men with OA. The differential expression of key genes and related signalling pathways might be associated with sex differences in the prevalence and symptoms of OA. However, the role of sex differences in OA is still understated (Li et al., 2022a). The orthopaedic community should pay more attention to sex differences when studying the pathogenesis and treatments of OA, especially in clinical trials.

### 2.4 Current therapeutic strategy and progress of osteoarthritis

As no disease-modifying treatments exist, total knee replacement or hip replacement is often considered an effective strategy for treating end-stage OA patients (Carr et al., 2012; Chen et al., 2021a), and the incidence of total knee arthroplasty continues to rise (Inacio et al., 2017). In addition, a recent study (Beard et al., 2019) reported that total knee replacement and partial knee replacement are both effective with similar clinical outcomes, but partial knee replacement has lower costs and better cost-effectiveness, which suggested that partial knee replacement should be considered the first choice for late-stage OA patients. Before surgical treatments, nonsteroidal anti-inflammatory drugs (NSAIDs) are first-line nonsurgical treatments for OA patients (Bannuru et al., 2019; Katz et al., 2021). For patients who are not recommended to take NSAIDs, intra-articular injections of corticosteroids or hyaluronic acid are primary options, which could relieve OA pain in the short term (Cheng et al., 2012; Bannuru et al., 2019). However, pharmacological therapies usually have serious side effects, including gastrointestinal irritation, bleeding, and decreased renal blood flow (Oray et al., 2016; Katz et al., 2021).

Therefore, many groups are dedicated to investigating the underlying mechanism of OA pathogenesis and exploring better pharmaceutical therapeutic targets to treat OA. For example, Li et al. (2020) reported that metformin had a chondroprotective effect to decelerate OA development and progression by enhancing AMPK expression and phosphorylation. As mentioned above, dietary cholesterol and accordingly increased plasma cholesterol levels play a role in the development of OA. Gierman et al. (2014) found that atorvastatin could significantly suppress OA development by improving cholesterol metabolism. Based on a mouse model of obesity, Deng et al. (2018) reported that eplerenone, a mineralocorticoid receptor antagonist usually used for diabetes, has a great therapeutic benefit on metabolic syndrome-related OA. Thorup et al. (2020) also reported that blocking receptor tyrosine kinase–like orphan receptor 2 can improve cartilage integrity and pain in mouse models. Qin et al. (Zhang et al., 2014; Wei et al., 2021b) found that the epidermal growth factor receptor (EGFR) pathway can be targeted to treat OA pathogenesis. They developed polymeric micellar nanoparticles conjugated with transforming growth factor-α, a potent EGFR ligand, which could attenuate surgery-induced OA cartilage degeneration. In addition, Elijah et al. (Carlson et al., 2021) suggested that paroxetine is a disease-modifying drug for OA that inhibits G protein-coupled receptor kinase 2. These findings provide promising pharmaceutical therapeutic targets for OA.

Although there have been great advances in pharmacological therapy for OA patients in recent decades, pharmacological treatments are sometimes associated with significant adverse side effects, and more research is required to evaluate the reliability and effectiveness of the new drug targets described above in OA patients. However, as a nonpharmacological and nonsurgical therapy, physical therapy is safe and easy to apply and may be beneficial for metabolism. In addition, previous studies have already demonstrated the great benefits of physical therapy for improving symptoms of OA (Deyle et al., 2000; Deyle et al., 2005; Rhon et al., 2022) (Figure 1).

## 3 Physical therapy of osteoarthritis

### 3.1 Physical therapies for osteoarthritis prevention and treatment

**FIGURE 2:**
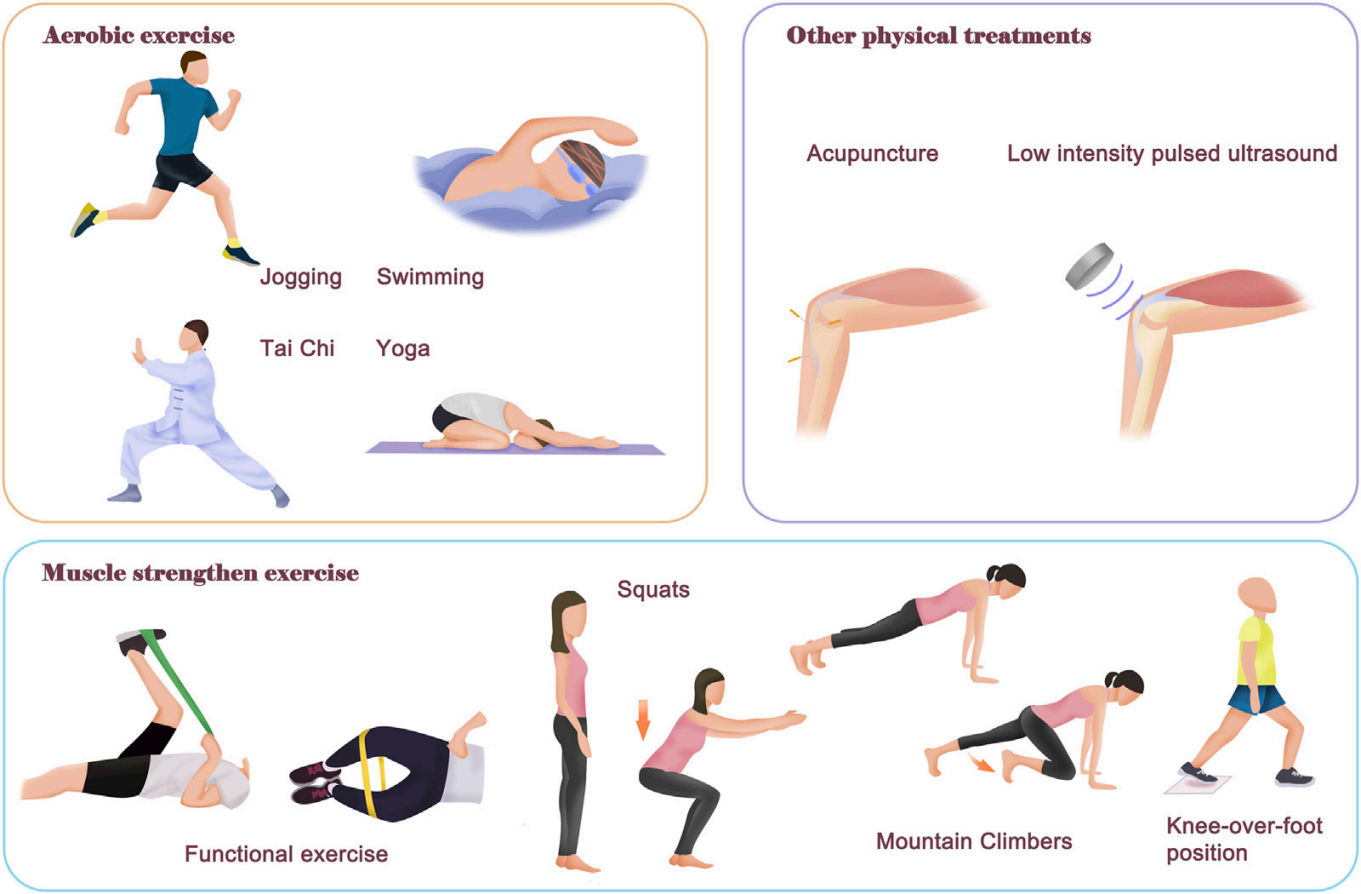
Contents of different physical therapies. The physical therapy for OA mainly includes aerobic exercise and neuromuscular exercise. The purpose of aerobic exercise is to improve body fitness, muscle strength and improve body metabolism. There are many different models of aerobic exercise, such as swimming, jogging, Tai Chi and yoga. The neuromuscular exercise aims to improve joint function and stability, as well as relieve symptom pain of OA patients. In addition, there are also other helpful physical therapies that rely on external instruments, such as acupuncture and Low intensity pulsed ultrasound treatment, which can relieve symptom pain and improve joint function. Notably, most of the time, a combination of physical therapies is required for better therapeutic effectiveness.

As mentioned above, OA is the most common joint disease and one of the leading causes of pain and disability worldwide, yet there are no disease-modifying drugs. Physical therapy represented by regular exercise has many advantages when compared with surgery and pharmacological intervention, such as ease of application, few adverse effects and relatively low costs. Therefore, physical therapy has unanimously been recommended as an important treatment strategy for OA by leading international organizations and authorities (Fernandes et al., 2013; Bannuru et al., 2019; Kolasinski et al., 2020) (Figure 2).

#### 3.1.1 Preclinical research

Many studies in animal models have demonstrated that physical therapy represented by exercise is beneficial in alleviating OA symptoms. For example, in a rat OA model induced by intra-articular monosodium iodoacetate, Joshua et al. (Allen et al., 2017) found that 4 weeks of treadmill exercise could induce pain relief in NSAID-resistant OA, likely dependent on endogenous opioid signalling. However, treadmill exercise did not alter radiographic evidence of OA but blocked monosodium iodoacetate-induced subchondral bone loss, which has been reported as a primary inducing reason for OA pain (Zhu et al., 2019). In another study, this group (Cormier et al., 2017) also reported that 21 days of prior voluntary wheel running can attenuate pain scores in rats, and the effect varies as a function of prior exercise duration. Interestingly, they also found voluntary wheel running-induced pain relief was associated with increased trabecular bone volume and thickness.

In addition, physical therapy can be beneficial for improving the main risk factors for OA. Hao et al. (2022) reported that 8 weeks of treadmill walking (15 m/min, 30 min/d, 5 days/week for 8 weeks) was effective at maintaining the integrity of cartilage-subchondral bone units in posttraumatic induced OA models. The amelioration effect of exercise is potentially involved in the modification of disease-relevant microbial shifts and microbiome metabolites. Many metabolic disorder-related OA models can also benefit from physical therapy. Fahaid et al. (Al-Hashem et al., 2017) reported that swimming exercise could protect the articular cartilage in mice with diabetes-induced OA. Li et al. (2022b) also found that the combination of metformin and exercise ameliorated the abnormal metabolic status and articular degeneration, but the underlying mechanisms were not fully understood. Park et al. (2022) reported that aerobic exercise could alleviate OA pain and articular cartilage degradation in testosterone-deficient OA rats by improving body metabolism, including decreased fat mass and lipid peroxide. In addition, physical therapy usually has good effects on protecting cartilage and decreasing systematic inflammation. Zhang et al. (2021) found that swimming exercise once a day (15 min/time) for 4 consecutive weeks could inhibit caspase-3 levels and chondrocyte apoptosis, thus improving joint morphology in OA rat models. Wheel exercise could also protect against inflammation in articular cartilage by down regulating the expression of inflammatory cytokines (Chen et al., 2020b).

Although OA symptoms can be improved by physical therapy, excessive exercise sometimes may lead to excessive mechanical stress on the joint and even promote OA progression. For example, Nils et al. (Bomer et al., 2016) reported that mice subjected to a forced running regime had significantly increased cartilage damage. Therefore, an appropriate intensity of exercise is critical to the effectiveness of physical therapy.

#### 3.1.2 Clinical studies

In recent decades, multiple clinical trials and meta-analysis from different groups have also investigated the effects of various diagrams of physical therapy on the symptoms of OA patients (Chen et al., 2021b; Vassão et al., 2021; Kalo et al., 2022). Trial sequential analysis and network meta-analysis from Olalekan et al. (Uthman et al., 2013) analyzed 60 trials covering 12 exercise interventions and 8218 patients, which showed that as of 2002 sufficient evidence supported the significant benefits of exercise over no exercise in OA patients. They suggested that an approach combining exercises to increase strength, flexibility, and aerobic capacity might be more effective in the management of lower limb OA. Many clinical trials from other groups have also demonstrated the effectiveness of various physical therapies. A clinical trial from Filiz et al. (Kılıç et al., 2020) showed that postmenopausal OA patients had superior OA-specific physical performance after 6 weeks of aerobic exercise (treadmill walking). Han et al. (2021) compared the long-term efficacy between low-intensity pulsed ultrasound combined with passive stretching exercise and exercise alone in a randomized controlled pilot study with 62 OA patients. They found that participants who received low-intensity pulsed ultrasound combined with passive stretching exercise achieved better efficacy in pain relief and knee function than those who exercised alone. Allen et al. (2021) also reported that a stepped exercise program resulted in better improvements in knee OA symptoms compared with the education-only group in a randomized controlled trial with 345 OA patients. A randomized clinical trial from Bokaeian et al. (2021) reported that yoga exercises and medial-thrust gait resulted in significant improvement in pain and function at the 1-month follow-up when compared with hamstring-strengthening exercise and treadmill walking. A recent study from Goh et al. (2019) systematically compared the relative efficacy of different exercises for pain, joint function, performance, and quality of life in 9134 OA patients from 103 clinical trials. According to their results, aerobic exercise (e.g., swimming, jogging) was most beneficial for pain and performance. Mindfulness exercise (e.g., Tai Chi, yoga) had equivalent pain benefits as aerobic exercise and better functional improvements. Strengthening exercises (e.g., lifting dumbbells, squats), flexibility exercises (e.g., hamstring stretch, gastrocnemius stretch) and neuromotor skills (e.g., wobble board, walking on foam) improved multiple OA symptoms at a moderate level. Surprisingly, mixed exercise was the least effective and the reasons remain unknown. However, although physical therapy has been considered as beneficial for OA patients, Elisabeth et al. (Bandak et al., 2021) suggested that none of the numerous randomized controlled trials has used adequately designed placebo comparison controls due to the lack of an underlying mechanism of exercise and education programs works on symptoms. Thus, future studies should take contextual factors into account when estimating treatment responses to physical therapy in OA.

Physical therapy is also generally thought to be important for OA patients before and after knee arthroplasty to achieve optimal outcomes, although the paradigm of rehabilitation varies (Hamilton et al., 2019; Smith et al., 2020). Previous meta-analyses suggested that physical therapy offers short-term benefits for all patients after total knee arthroplasty but has limited effecacy at 1 year (Minns Lowe et al., 2007; Artz et al., 2015). Usually, increased physiotheist contact is thought to enhance the rehabilitation of OA patients after knee arthroplasty, especially prolonging postoperative inpatient rehabilitation. For instance, a quasi-experimental study form Dimitrios et al. (Vasileiadis et al., 2022) demonstrated that a 6-week physical therapy program supervised by a physiotherapist before surgery is efficacious for decreasing pain, improving knee function, and enhancing living activities. However, recent studies suggested that inpatient or clinic-based rehabilitation offers no clinically important advantages when compared with home-based rehabilitation (Buhagiar et al., 2019). A randomized controlled trial from David et al. (Hamilton et al., 2020) also reported that the actual content of the rehabilitation seems to have minimal influence on patient outcomes, but targeting rehabilitation interventions to at-risk patients is a feasible delivery method.

As a traditional Chinese physical therapy, acupuncture is safe, with few or minor adverse events, and has been recommended to treat patients with musculoskeletal system disorders. A network meta-analysis from Corbett et al. (2013) indicated that acupuncture can be considered one of the more effective physical treatments for alleviating osteoarthritis knee pain in the short term. However, Nadine et al. (Foster et al., 2007) conducted a randomized controlled trial and reported that the addition of acupuncture to a course of exercise for knee OA patients provided no additional improvement in pain scores. Acupuncture is a complex intervention, and its nature is not fully understood, which may lead to inconsistent results from different groups. Its effects are subject to many factors, and one important factor is the selection of acupuncture manipulating points. Jia et al. (2020) reported that acupuncture at higher sensitized points is associated with improved clinical outcomes when compared to low/nonsensitized points. The inconsistent effects of acupuncture on OA may also be caused by the different acupuncture models and analyzed time points. Tu et al. (2021) reported that compared with the sham group, intensive electroacupuncture resulted in less pain and better function at week 8, and these effects persisted until week 26. However, intensive manual acupuncture had no beneficial effects for OA at week 8, but it showed benefits during follow-up.

Furthermore, there are many different types of physical therapies that are recommended for OA. Recently, a randomized clinical trial from Stephan et al. (Reichenbach et al., 2020) compared the effects of biomechanical footwear and control footwear on relieving OA pain. Their results showed that biomechanical footwear resulted in an improvement in pain at 24 weeks of follow-up that was statistically significant but of uncertain clinical importance. A cross-sectional study from Muollo et al. (2022) also found that the knee flexor torque and knee muscle quality decline with ageing and obesity, which emphasized that physiologists should include exercises designed to train both the knee flexor and knee extensor in elderly OA patients with obesity. However, some kinds of physical therapy should not be recommended for elderly OA patients. For instance, Orssatto et al. (2018) reported that 12 weeks of progressive training of a 45° leg press exercise (two sessions/week) could reduce the knee functional ratio (knee flexor torque/knee extensor torque) in elderly OA patients. Therefore, although there are many more different modalities of physical therapies that can be developed, more clinical trials need to be performed to determine the appropriate modality and intensity of physical therapy for different kinds of OA patients.

### 3.2 Future improvements of physical therapy in osteoarthritis treatments

#### 3.2.1 Patient education

As mentioned above, there is strong evidence that physical therapy represented by regular exercise can be beneficial for OA. However, inconsistent results are usually obtained by different exercise models and different research groups, mainly because of that most patients cannot strictly follow the requirements of physical therapies (Moseng et al., 2020). The low exercise adherence rate is one of the reasons why exercise therapy does not respond well in some cases (Østerås et al., 2019). In addition, there are many reasons for the low adherence of patients. The most common reason is that patients do not understand or are not interested in physical therapy. In addition, previous exercise behaviour and intact concepts of patients are also potential influencing factors. Moreover, the health status and jobs of patients may also determine their participation in exercise therapy. Thus, future studies should pay more attention to improving the education of OA patients to help them prolong adherence and optimize the effectiveness of physical therapy.

#### 3.2.2 Standard and personalized exercise modality

There is no denying that OA patients should receive physical therapy as early as possible to achieve the best curative effects. However, there are no unified standards of physical therapy for OA in clinical practice, leading to the inconsistency of the current clinical trials from different groups and the effectiveness of physical therapy, as well as the low acceptance of physical therapy for some OA patients.

Usually, to improve the adherence of patients to physical therapy, health education for patients and regular monitoring by clinical physiologists are strongly recommended in exercise prescription. Targeted assessment and professional exercise guidance can strengthen patients’ compliance with physical therapy and achieve the optimal effect. A randomized controlled trial from Kelli et al. (Allen et al., 2021) reported that a stepped exercise program showed modest improvements in the symptoms of knee OA patients. In this study, the stepped program intervention began with an internet-based exercise program. Participants who did not meet the response criteria for improvement in pain and function progressed to the next step, which involved 3 months of biweekly physical activity coaching calls. Then, participants who did not meet the response criteria after step 2 went on to in-person physical therapy visits. Thus, patient compliance with the prescriptions from clinicians or physiotherapists is of the most importance to the effectiveness of physical therapy, and physiotherapists should develop better ways to improve patient education.

Moreover, the selection of appropriate physical therapy content and intensity level also plays an important role in the effectiveness of physical therapy for OA (Aily et al., 2021; Wise, 2022). Therefore, more detailed and unified standards of physical therapy for OA are needed, by which clinicians or rehabilitation therapists can make personalized prescriptions according to the different situations. OA patients can also have a better understanding of the importance and principle of physical therapy based on the detailed standards or recommendations.

#### 3.2.3 Self-management programs

In addition, due to the growing burden of OA on the health system, the increasing need to ensure high-quality integrated services and the rapid advances in communication technology, self-management programs delivered through digital technologies could be an economical and effective model for physical therapy of OA (Slomski, 2021; Bennell et al., 2022).

In recent years, digital-based self-management programs have been proven to be an important component of physical therapy for OA patients, offering a sustainable opportunity to monitor patients’ symptoms for intervention adaptation and improve patient outcomes. Kroon et al. (2014) revealed that, compared with usual care control groups, self-management programs resulted in a significant but clinically unimportant reduction in pain up to 1 month postintervention. A recent meta-analysis (Safari et al., 2020) also reported that digital-based structured self-management programs moderately reduced pain and improved physical function compared to face-to-face interventions at the 12-month follow-up in OA patients.

However, there are also challenges with digital programs that have limited practitioner support, including poor adherence and high dropout rates. Rhiannon et al. (Patten et al., 2022) suggested that digital interventions designed for a targeted condition are a promising approach for promoting high adherence and reducing attrition. Future studies should further compare the adherence of digital interventions with face-to-face interventions and develop strategies to promote long-term adherence.

#### 3.2.4 Combined physical therapy with other treatments

As an adjunct treatment, physical therapy may work better in combination with other treatments to optimize the effectiveness for OA patients, such as combined with a diet intervention (Hall et al., 2022), pain-reliving drugs (Soriano-Maldonado et al., 2016; Riis et al., 2017) and other treatments.

Pharmacological therapy mainly aims to relieve pain and inflammation in OA patients, but anti-inflammatory treatment before physical therapy may also enhance the effects of exercise. Thus, a combination of pharmacologic and physical therapy modalities is recommended for the optimal management of OA (Jordan et al., 2003; Rausch Osthoff et al., 2018). However, few studies have investigated whether such combination therapy would provide better clinical benefits. Marius et al. (Henriksen et al., 2015) conducted a randomized clinical trial to assess the clinical benefits of an intra-articular corticosteroid injection given before exercise therapy in knee OA patients. Unfortunately, they observed no additional benefit of adding an intra-articular injection of corticosteroids before exercise in painful OA patients. The study from Alberto et al. (Soriano-Maldonado et al., 2016) obtained similar results that intra-articular corticosteroid injection 2 weeks prior to an exercise program does not provide additional benefits compared to placebo in reducing the pain sensitivity of OA patients. Another group (Riis et al., 2017) also reported that there were no statistically significant differences between intra-articular corticosteroid and placebo injections given before exercise therapy in regard to the reduction of synovitis in knee OA. Recently, a cohort analysis (Mantovani Cardoso et al., 2021) reported that patients who underwent corticosteroid injection before physical therapy were more likely to attend more sessions and have a longer adherence. Thus, a combination of pharmacologic and physical therapy modalities is still a promising treatment strategy, but further research is needed to establish optimal and potentially synergistic combinations of pharmacologic and physical therapy.

In recent years, many studies have confirmed that endogenous stem cells recruited by cytokines from inflammatory cells are an important source for tissue repair in the skeletal system (Lee et al., 2015; Ortinau et al., 2019), including the articular cartilage, which is usually damaged in OA. For example, Koelling et al. (2009) discovered a unique progenitor cell population in the repair tissue from the articular cartilage of OA patients, which may be relevant in the development of novel therapeutics for OA. Recently, Murphy et al. (2020) also demonstrated that a local expansion of stem cells could be triggered in the chondral surface after a microfracture surgery in mouse limb joints, and localized delivery of BMP2 coupled with soluble VEGFR1 in a hydrogel could induce resident stem cells to generate cartilage, offering a new strategy for OA treatment. As described above, endogenous stem cells are usually activated by various cytokines from inflammatory cells. However, few studies have investigated the effects of mechanical force or other physical stimuli of physical therapy on stem cells. The mechanical force or other stimuli of physical therapy may also activate stem cells, and a combination of physical therapy and stem cell therapy may improve their efficacy for OA treatment.

The continued advances in the field of biomaterials have triggered the great potential to enable restoration of damaged cartilage tissue in OA patients. Many studies have demonstrated the great efficacy of different biocompatible materials modified with various active ingredients on the treatment of OA in animal models (Maihöfer et al., 2021; Xie et al., 2021; Han et al., 2022). Moreover, combining mechanically stimulated signalling targets with the rapidly expanding field of mechanoresponsive biomaterials is an emerging paradigm in OA treatment (Hodgkinson et al., 2022). Interestingly, Yang et al. (Liu et al., 2022) recently developed a novel biodegradable piezoelectric poly (l-lactic acid) nanofibre scaffold, which could act as a battery-less electrical stimulator and generate a controllable piezoelectric charge under physical exercise, promoting extracellular protein adsorption, facilitating cell recruitment and improving cartilage regeneration. They found that rabbits with osteochondral defects receiving the piezoelectric scaffold and exercise treatment experienced hyaline cartilage regeneration and completely healed cartilage with abundant chondrocytes and type II collagen after 1–2 months of exercise. This approach of combining biodegradable scaffolds with controlled physical exercise may therefore be a promising model for the treatment of OA.

## 4 Conclusion

OA is the most prevalent chronic joint disease worldwide and is among the leading causes of pain and disability. Current pharmacotherapies for OA usually aim to alleviate symptoms by pain relief and anti-inflammatory drugs. In recent years, physical therapy has gradually been recommended as a nonpharmacological treatment for OA due to its cost savings, convenience, and few adverse reactions. As an adjunctive therapy, physical therapy aims to relieve pain, improve joint function, and improve the daily living quality of OA patients. Physical therapy interventions for OA patients vary worldwide and mainly include muscle strength training, aerobic exercise training, resistance training, and yoga and Tai Chi. However, the effectiveness of physical therapy on OA has not been fully investigated, nor has the mechanism by which physical therapy improves OA symptoms. Therefore, more research and clinical trails are needed to clarify the effect of physical therapy on OA.

In addition, there are no unified standards for the indications, contraindications, optimal treatment parameters and optimal course of physical therapy in clinical practice, which not only hampers the clinical effectiveness of physical therapy for OA but also leads to inconsistences in the current clinical trials from different groups. Besides, the subjective initiative of patients is also an important factor affecting the consistency of clinical trials. Further studies should focus more on improving patients’ education to help them fully understand the benefits of physical therapy and prolong adherence. Moreover, more studies should also be considered to investigate the different combinations of pharmacologic and physical therapy modalities for optimal management of OA. Thus, it is important to formulate a more detailed and unified standard of physical therapy for OA, by which clinicians or rehabilitation therapists can make personalized prescriptions according to the different situations of OA patients at different stages.

## 5 Contribution

In this review, we first introduced the prevalence, symptoms, and diagnosis of OA and then discussed the risk factors for OA in detail, which could not only give readers a comprehensive understanding of this degenerative disease but also help clinicians understand that OA is a systemic and multiorgan disease that should be considered from several aspects. Next, we discuss the limitations of current clinical treatments for OA, which mainly include pharmaceutical therapy and surgical therapy. However, there are some limitations. The advantages and disadvantages of different medications and operations are not reviewed in detail in this part. However, we emphatically reviewed recent studies of physical therapy in the treatment of OA and discussed the present challenges and promising development directions of physical therapy, which can help physiotherapists develop better modalities of physical therapies in the future.
